# Functional detection of botulinum neurotoxin serotypes A to F by monoclonal neoepitope-specific antibodies and suspension array technology

**DOI:** 10.1038/s41598-019-41722-z

**Published:** 2019-04-02

**Authors:** Laura von Berg, Daniel Stern, Diana Pauly, Stefan Mahrhold, Jasmin Weisemann, Lisa Jentsch, Eva-Maria Hansbauer, Christian Müller, Marc A. Avondet, Andreas Rummel, Martin B. Dorner, Brigitte G. Dorner

**Affiliations:** 1 Biological Toxins (ZBS 3), Centre for Biological Threats and Special Pathogens, Robert Koch Institute, Berlin, 13353 Germany; 2 Institut für Toxikologie, Medizinische Hochschule Hannover, 30625 Hannover, Germany; 3 Spiez Laboratory, Federal Office for Civil Protection, Spiez, 3700 Switzerland; 4 Department of Ophthalmology, University Hospital Regensburg, Regensburg, 93053 Germany

## Abstract

Botulinum neurotoxins (BoNTs) are the most potent toxins known and cause the life threatening disease botulism. Sensitive and broad detection is extremely challenging due to the toxins’ high potency and molecular heterogeneity with several serotypes and more than 40 subtypes. The toxicity of BoNT is mediated by enzymatic cleavage of different synaptic proteins involved in neurotransmitter release at serotype-specific cleavage sites. Hence, active BoNTs can be monitored and distinguished *in vitro* by detecting their substrate cleavage products. In this work, we developed a comprehensive panel of monoclonal neoepitope antibodies (Neo-mAbs) highly specific for the newly generated N- and/or C-termini of the substrate cleavage products of BoNT serotypes A to F. The Neo-mAbs were implemented in a set of three enzymatic assays for the simultaneous detection of two BoNT serotypes each by monitoring substrate cleavage on colour-coded magnetic Luminex-beads. For the first time, all relevant serotypes could be detected in parallel by a routine *in vitro* activity assay in spiked serum and food samples yielding excellent detection limits in the range of the mouse bioassay or better (0.3–80 pg/mL). Therefore, this work represents a major step towards the replacement of the mouse bioassay for botulism diagnostics.

## Introduction

Botulinum neurotoxins (BoNTs) represent the most poisonous biological substances known today and are the causative agents of the rare but severe neurological disease botulism in humans and animals^1,2^. In humans, botulism can be caused by ingestion of food contaminated with the toxins (food-borne botulism). Uptake and outgrowth of bacterial spores may lead to infant or wound botulism^3^. Botulism is characterised by descending flaccid paralysis due to a blockage of neurotransmitter release at neuromuscular junctions, which potentially leads to death due to respiratory failure. The exclusive neurospecificity and paralysing effect of BoNT makes it an effective therapeutic against a broad range of neurological and non-neurological diseases and in aesthetic medicine^4,5^.

On the molecular level, BoNTs are a highly variable group of toxins produced by ubiquitously occurring Gram-positive, spore forming, anaerobe *Clostridium* spp. (*C. botulinum* groups I–IV, *C. baratii*, and *C. butyricum*). Until now, seven widely accepted “classical” BoNT serotypes designated BoNT/A–G are known of which six naturally induce botulism in humans (BoNT/A, B, E, F) or animals (predominantly BoNT/C and D and their mosaic variants CD and DC)^6–9^. BoNT/G has not been clearly assigned to a natural outbreak in humans or animals^9^. Additionally, several novel BoNT molecules have been pronounced: BoNT/HA (also called BoNT/FA or BoNT/H) which was involved in an infant botulism case and has been characterised as a novel mosaic type toxin^10–12^ and BoNT/X,^13^ both produced or encoded by *C. botulinum,* as well as eBoNT/J (aka BoNT/En) encoded by *Enterococcus faecium*^14–16^. Advanced whole genome sequencing technologies revealed differences within a given serotype for BoNT/A, B, E and F leading to the introduction of subtypes and adding further variability to the BoNT family^6^. The subtypes can differ up to 36% on the amino acid level within a given serotype. So far, more than 40 subtypes have been described in the literature^9^. Subtypes of a given serotype have been shown to differ in their biological activity, *e.g*. their kinetics of uptake and substrate cleavage, affinity to receptors or overall activity^17–21^.

**Figure 1:**
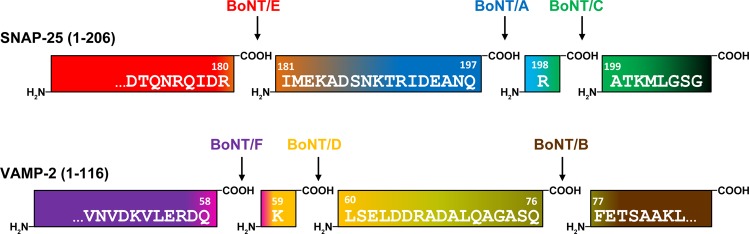
Cleavage sites of BoNT serotypes pathogenic to humans or animals in SNAP-25 or VAMP-2. Serotypes can be distinguished by their substrate specificity and specific cleavage position. After cleavage, neo-epitopes arise on both the C- and N-terminal ends of the cleavage sites that can be detected by highly specific neo-epitope antibodies.

The mechanism of action is mediated by different domains of the toxin: BoNT is produced as a 150 kDa holotoxin which, upon proteolytic activation, is cleaved into a 100 kDa heavy chain (HC) and a 50 kDa enzymatically active light chain (LC) which remains connected to HC by a single disulfide bond^22^. The HC consists of a 50 kDa C-terminal receptor binding domain (H_C_) and a 50 kDa translocation domain (H_N_). BoNT exerts its extreme potency by binding to specific neuronal receptors mediated via its H_C_ domain, subsequent uptake into recycling endosomes followed by H_N_-mediated translocation of the LC into the cytosol, and finally LC-mediated cleavage of soluble N-ethylmaleimide-sensitive-factor attachment receptor (SNARE)-complex proteins thereby blocking neurotransmitter release^23–27^. Here, different BoNT serotypes cleave different SNARE proteins at distinct sites: synaptosomal-associated protein of 25 kDa (SNAP-25) is cleaved by BoNT/A, C, and E while vesicle-associated membrane protein 2 (VAMP/synaptobrevin-2) is cleaved by BoNT/B, D, F, and G (Fig. 1). BoNT/C additionally cleaves syntaxin and is therefore the only classical serotype that hydrolyses two substrates^28^. Each serotype has a unique cleavage site on the respective substrate molecule, indicating that the presence of different BoNT serotypes can be detected by analysis of the cleavage products generated^26,29^. Notably, different subtypes of a given serotype target the very same cleavage site on the respective substrate with one known exception: BoNT/F5 uses a different cleavage site in VAMP-2 than all other BoNT/F subtypes; the same cleavage site is also targeted by the recently identified BoNT/HA^11,30^.

Because of the large variability of BoNTs on the molecular level, its detection is highly challenging. Due to its high potency minute amounts in the low pg per mL range of all different BoNT serotypes must be detected reliably in complex clinical, food or environmental samples^31,32^. Based on these facts the mouse bioassay (MBA) is still considered the gold standard for diagnostics of botulism. Here, particle-free sample material (*e.g*. serum, bacterial cultures, homogenised food matrices) diluted in buffer is injected intraperitoneally into mice which are subsequently monitored for up to four days for clinical signs of botulism such as laboured breathing, a wasp-like narrowed waist due to increased respiratory efforts, weakness of limbs that progress to total paralysis and finally death by respiratory failure^31,33^. The disease causing serotype is determined by toxin-neutralisation employing serotype-specific antibodies which increases the number of animals needed. The MBA is highly sensitive with a 50% lethal dose (LD_50_) in mice corresponding to 5 to 50 pg per mouse for BoNT/A, B, E and F, respectively^34,35^, reflecting the pharmacokinetics and pharmacodynamics, the latter comprising receptor binding, uptake, translocation and enzymatic activity of all serotypes. Additionally, the MBA can detect all ‘mouse-toxic’ sero- and subtypes (including any yet unknown subtypes) out of complex matrices, which is crucial for reliable diagnostics. However, drawbacks are long assay duration of up to four days, considerable costs associated with animal husbandry, several technical limitations such as species differences between mice and humans^36–38^ and, importantly, serious ethical concerns due the suffering inflicted on the animals^33^.

Therefore, alternative assays to replace the MBA have long been pursued^31–33,39,40^. Of those, endopeptidase assays monitor the toxin’s catalytic activity, which displays the major determinant of BoNT toxicity. A highly sophisticated approach successfully employing this principle has been established for BoNT/A to G by monitoring cleavage products of short serotype-specific peptide substrates by mass spectrometry (MS; Endopep-MS assay)^41,42^. While the Endopep-MS assay allows for the sensitive detection of all known serotypes with high confidence, the applicability of this approach is limited to expert laboratories due to the high level of expertise and expensive technical instrumentation needed. For a broader application, substrate peptides labelled with flanking fluorescence donor and quencher molecules have been designed to measure the catalytic activity of BoNT by Förster resonance energy transfer (FRET)^43–49^. Advantages of FRET-based approaches are their high sensitivity combined with a simple and fast assay protocol. Furthermore, commonly available laboratory equipment is sufficient to perform the assays ensuring a broad applicability in routine laboratories. A major drawback, however, is that serotypes targeting the same substrate cannot be distinguished by this method, since cleavage at every site between donor and acceptor fluorophore generates a signal. Shorter peptide substrates enabling a discrimination of serotypes E or A/C and B or F/D may be applied, but presumably lead to a loss in sensitivity due to the lack of important exosites^49^. Still, the discrimination of cleavage by BoNT/A and C or BoNT/F and D is not possible without further substrate modifications, since their respective cleavage sites on SNAP-25 or VAMP-2 are only one amino acid apart (Fig. 1). Noteworthy, cleavage of the substrate peptide used in FRET endopeptidase assays by other proteases present in the sample leads to false positive results. To develop straightforward assays capable of discriminating between all BoNT serotypes, neoepitope specific antibodies (Neo-Abs) have been introduced. Per definition, Neo-Abs exclusively recognise the newly exposed epitopes in the substrate molecules SNAP-25 or VAMP-2 after cleavage by a given BoNT, but do not recognise the intact, uncleaved substrate or substrate cleaved at a distinct position^50^. Different assay platforms employing polyclonal Neo-Abs have been proposed for the detection of BoNT/A, B, C, D, E, and F^51–57^. However, polyclonal Neo-Abs have major drawbacks for routine diagnostics: they imply the risk of cross-reactivity against uncleaved substrate as well as neighbouring cleavage sites, especially in case of BoNT/A and C or BoNT/D and F, both separated by one amino acid. In addition, detection of BoNT in complex matrices may be hampered by interferences of the polyclonal Neo-Abs with matrix components^58,59^. Due to their higher specificity monoclonal antibodies can overcome these limitations but their generation is challenging, especially for reagents targeting directly adjacent cleavage sites. So far, assays using monoclonal neoepitope specific antibodies (Neo-mAbs) have been described for BoNT/A and E only^60–65^.

In this work, we generated a unique and comprehensive panel of monoclonal antibodies directed against neoepitopes of VAMP-2 or SNAP-25 after cleavage by BoNT/A, B, C, D, E and F. Out of 20 Neo-mAbs generated, six outperforming antibodies (one for each serotype BoNT/A to F) were selected and implemented in a novel functional suspension array based on the Luminex platform^66^: The Neo-mAbs were implemented in a set of three enzymatic assays for the simultaneous detection of two BoNT serotypes each by monitoring substrate cleavage on colour-coded magnetic Luminex-beads. After toxin enrichment using monoclonal antibodies coupled to paramagnetic beads the assays were capable of detecting enzymatically active toxin with detection limits of 0.3–13 pg/mL for BoNT/A, B, E and F and 79.1 pg/mL and 1.1 pg/mL for BoNT/C and D, respectively, when testing toxin-spiked buffer, serum or food. Based on its sensitivity and specificity, the novel functional suspension array represents a major advancement in BoNT diagnostics with the potential to replace the MBA in routine applications.

## Results

### Generation of highly specific monoclonal neoepitope specific antibodies

A major aim of this study was to develop a comprehensive panel of Neo-mAbs targeting the newly generated N- or C-termini in the cleavage products of SNAP-25 or VAMP-2 after proteolysis by BoNT/A to F. This task was especially challenging for the serotypes BoNT/A and C as well as BoNT/D and F which target adjacent peptide bonds in SNAP-25 or VAMP-2, resulting in cleavage sites separated by only one amino acid (Fig. 1). Mice were immunised with BSA-coupled 8-mer peptides corresponding to the new N- or C-terminal sequence of the respective cleavage products to induce antibodies restricted to the eight terminal amino acids adjacent to the cleavage site (Fig. 1). Hybridoma clones were subjected to a stringent screening procedure to ensure that only the cleavage products but neither the uncleaved substrate nor the cleavage products of a different serotype were detected.To this aim we tested binding towards the specific peptides as well as peptides covering the other cleavage sites at the same substrate by indirect ELISA. Those peptides were coupled to KLH to exclude antibodies recognising the carrier protein BSA. Furthermore, binding to intact SNAP-25 or VAMP-2 was tested to exclude cross-reactive antibodies from further characterisation. Overall, more than 20,000 hybridoma clones generated in 10 fusions were tested for production of Neo-mAbs. Eventually, 20 promising Neo-mAbs were identified based on their high reactivity against specific peptides targeting the different cleavage products of SNAP-25 and VAMP-2 after proteolysis by BoNT/A to F, respectively, and the lack of cross-reactivity against intact substrate (Table 1 and Fig. S1).

**Table 1:** Overview of all Neo-mAbs generated in this work for the detection of catalytically active BoNT.

Serotype	Antibody^a^	Isotype	Specificity^b^	Endopep-ELISA^c^
BoNT/A	**SNAP/A/291**	**IgG1**	**N**	+++
	SNAP/A/305	IgG1	N	+++
BoNT/B	**VAMP/B/1148**	**IgG1**	**N**	+++
	VAMP/B/226	IgG1	N	+++
	VAMP/B/151	IgG2b	C	+
	VAMP/B/392	IgG1	C	−
	VAMP/B/726	IgG3	C	−
BoNT/C	SNAP/C/2207	IgG1	C	−
	**SNAP/C/5593**	**IgG2a**	**N**	+++
	SNAP/C/1844	IgG2a	N	−
	SNAP/C/3280	IgG2b	N	−
BoNT/D	**VAMP/D/27**	**IgG2b**	**C**	+++
	VAMP/D/29	IgG2b	C	++
BoNT/E	SNAP/E/1466	IgG1	C	−
	**SNAP/E/217**	**IgG1**	**N**	**+**
BoNT/F	VAMP/F/440	IgG2a	C	+++
	VAMP/F/153	IgG1	N	−
	VAMP/F/521	IgG1	N	+++
	**VAMP/F/425**	**IgG1**	**N**	**+++**
	VAMP/F/1333	IgG1	N	+++

^a^Antibodies depicted bold were selected for implementation into the functional Luminex suspension array.

^b^Specificity of Neo-mAbs towards N- or C-terminal fragment generated after cleavage by indicated BoNT. ^c^EC_50_ in Endopep-ELISA: + + + = < 0.1 ng/ml; + + = < 1 ng/ml; + = < 10 ng/ml; −  = signal too weak for detection.

To select antibodies most suitable for the implementation in a functional suspension array we compared the performance of all 20 mAbs in an endopeptidase cleavage ELISA (Endopep-ELISA) as described previously by Jones *et al*.^55^. VAMP-2 or SNAP-25 was coated on microtitre plates, hydrolysed by serial dilutions of BoNT/A to F and cleavage products were detected by Neo-mAbs. As shown in Fig. S2, remarkable differences in performance were observed for the 20 Neo-mAbs in Endopep-ELISA, although some mAbs were seemingly equivalent in their recognition of 8-mer peptides representing individual neo-epitopes (Fig. S1, *e.g*. mAb VAMP/D/27 and VAMP/D/29). Based on the Endopep-ELISA results six Neo-mAbs showing superior performance – one for each serotype – were selected for a more detailed characterisation (indicated in bold in Table 1).

**Figure 2:**
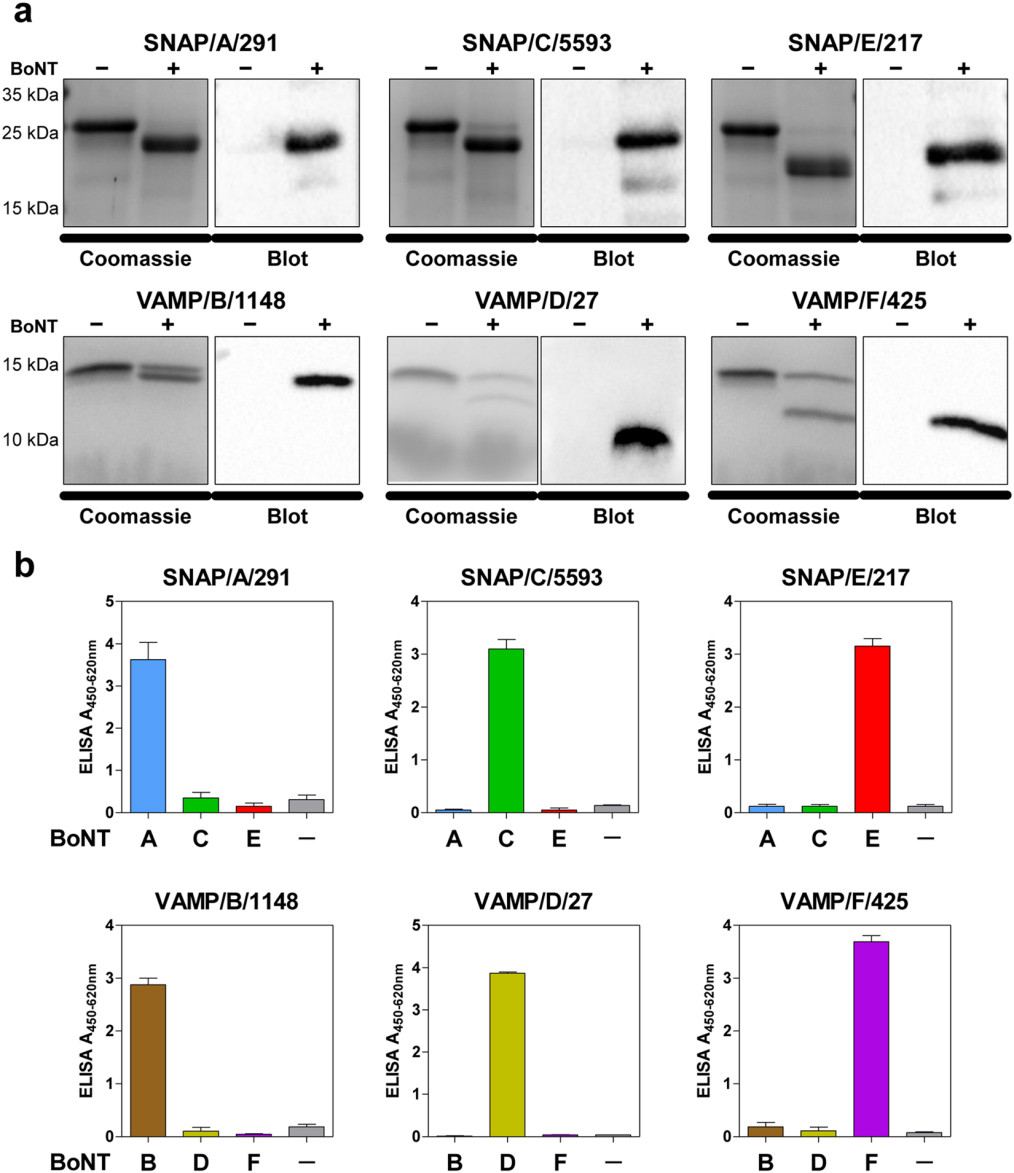
Selected neoepitope specific monoclonal antibodies exclusively recognise the specific cleavage site of the corresponding BoNT. (**a**) Western blot results to compare detection of cleaved vs. uncleaved SNAP-25 or VAMP-2 by the Neo-mAbs. SNAP-25 or VAMP-2 was incubated with (+) or without (−) BoNT overnight at 37 °C in cleavage buffer, samples were loaded on two identical polyacrylamide gels of which one gel was used for Coomassie staining, and the second gel was applied for Western blotting using Neo-mAbs. (**b**) Endopep-ELISA demonstrating specific recognition of cleavage sites. VAMP-2 or SNAP-25 were coated on microtitre plates, cleaved with 10 ng/mL BoNT as indicated and cleavage products were detected with Neo-mAbs indicated above each panel. Results from two independent experiments, each performed in technical duplicates are shown (n = 4; mean ± SD).

We first tested specificity towards cleaved but not uncleaved substrate by Western blotting. To this aim, BoNT-cleaved and intact VAMP-2 or SNAP-25 were analysed by SDS-PAGE, Coomassie staining and Western blotting. Here, all six antibodies exclusively recognised the cleaved, but not the intact substrate (Fig. 2a). A crucial requirement of the selected Neo-mAbs was their specific recognition of only one cleavage site, while cross-reactivity towards adjacent cleavage sites had to be excluded. We therefore tested each antibody against substrates cleaved with high concentrations (10 ng/mL) of the next-neighbour BoNT molecules by Endopeptidase-ELISA. Importantly, the six antibodies were exclusively specific for a single neoepitope in cleavage products of SNAP-25 or VAMP-2, allowing the clear discrimination of catalytic activity of BoNT/A to F. This was particularly notable for BoNT/A and C as well as for BoNT/D and F, since the cleavage sites of these serotypes differ by only one amino acid position (Fig. 2b). Hereby, much of the complexity associated with the comprehensive detection of all known and yet unknown subtypes within each serotype is reduced to detection of only six enzymatically active “cleavotypes”^9,32^.

The six selected Neo-mAbs were further used for setting up a novel Luminex-based suspension array for the detection of enzymatically active BoNT/A to F.

### Establishment of three Luminex duplex-assays for the simultaneous detection of SNAP-25- and VAMP-2-targeting serotypes

#### Assay principle and setup

The primary goal of this work was to establish an assay that enables the detection of BoNTs from complex matrices in a routine diagnostic setting as an alternative to the mouse bioassay. Previous works had encountered matrix interferences when using cleavage based assays for the analysis of complex clinical or food samples (*e.g*. due to unspecific proteases or incompatible buffer requirements^31,61,67^). Therefore, a toxin enrichment step was included prior to the Luminex assay to enrich the toxins and to provide optimum buffer conditions for subsequent substrate cleavage. To this end, paramagnetic beads were coupled with monoclonal anti-BoNT antibodies generated in our laboratory targeting the H_C_ or H_N_ domains of BoNT/A to F (Supporting methods, Table S1, Fig. S3). By using the toxins’ receptor-binding or translocation domains for enrichment a potentially negative influence on the enzymatic activity by antibodies targeting the LC was excluded^68^.

To enable the detection of all clinically relevant BoNT serotypes with minimal sample consumption we implemented the six candidate Neo-mAbs in a novel enzymatic multiplex suspension assay using the Luminex platform. This system uses colour-coded paramagnetic Luminex beads, thereby enabling the detection of multiple analytes in one sample. Initially, we were aiming at coupling the Neo-mAbs to the Luminex beads to capture only cleaved biotinylated SNAP-25 and VAMP-2 substrates from solution. This setup would have enabled a simultaneous detection of all six serotypes from one sample. However, this hexaplex approach failed, mainly due to unexpectedly strong unspecific binding of VAMP-2 substrate to the Luminex beads (Fig. S4a). Although this unspecific binding could be blocked efficiently by the addition of carboxymethyl-dextran, the overall sensitivity of this assay set-up was unsatisfactory (Fig. S4b).

**Figure 3:**
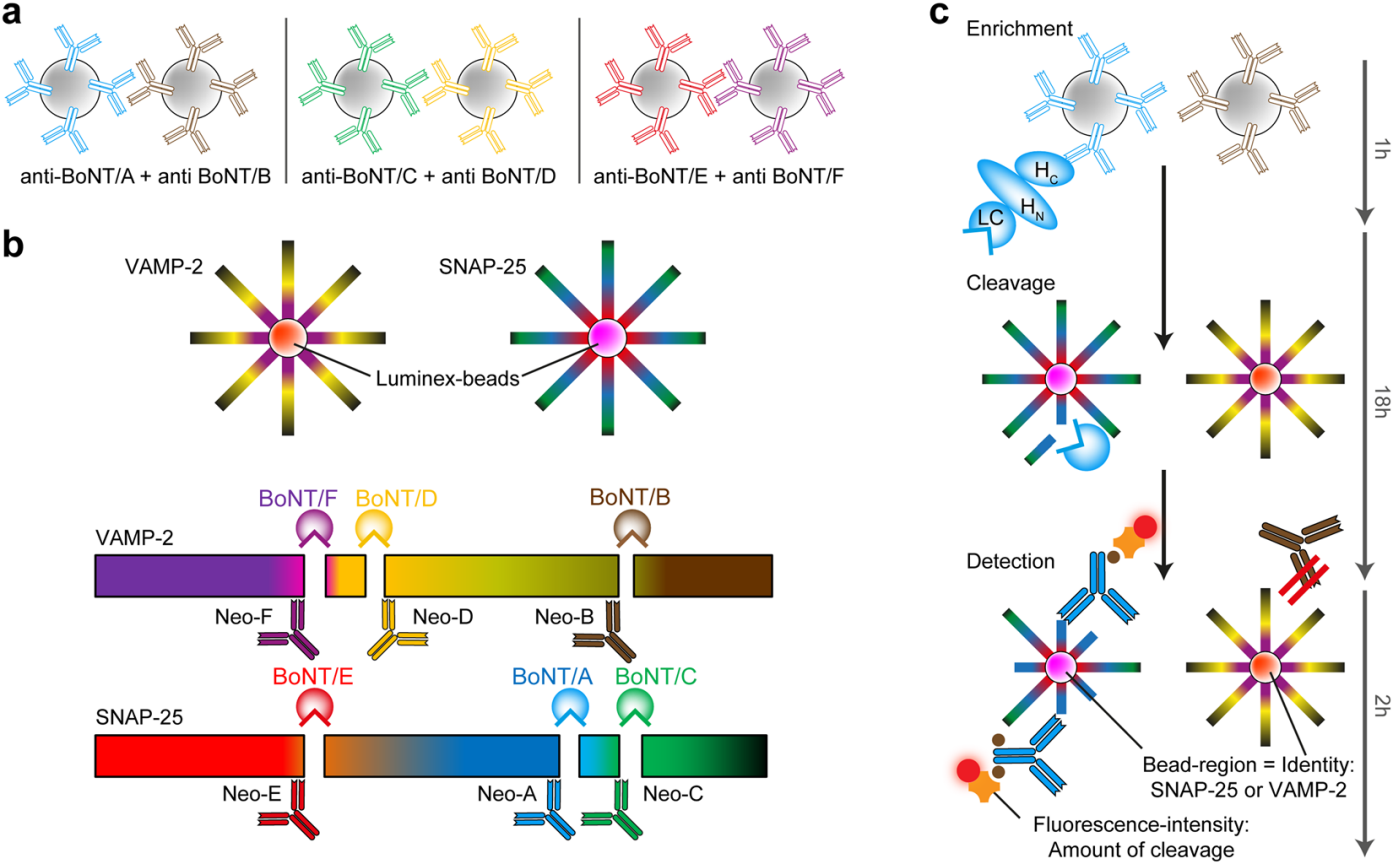
Schematic illustration of the Luminex duplex-assay working principle. (**a**) Paramagnetic beads coupled to monoclonal anti-BoNT antibodies are added to a toxin containing sample for toxin enrichment. For the three duplex-assays paramagnetic beads containing either anti-A and anti-B, or anti-C and anti-D, or anti-E and anti-F are mixed to enable simultaneous enrichment of two different serotypes in one sample. (**b**) SNAP-25 and VAMP-2 are coupled to two different regions of Luminex microspheres. Luminex microspheres are paramagnetic beads carrying carboxyl groups to enable coupling of molecules via primary amine groups. Each Luminex bead region carries a distinct ratio of two different fluorescent dyes. Hereby up to 100 colour-coded bead regions can be discerned via their fluorescence emission pattern after excitation. Different BoNT serotypes cleave SNAP-25 or VAMP-2 at distinct, individual sites. The resulting serotype-specific neoepitopes can be detected by Neo-mAbs (Neo-A to Neo-F). The differently coded microspheres for SNAP-25 and VAMP-2 allow for the simultaneous detection of one SNAP- and one VAMP-specific BoNT. (**c**) Workflow of the duplex-assay exemplarily shown for the BoNT/A + BoNT/B duplex assay with BoNT/A contained in the sample: After enrichment captured toxin is added to VAMP-2 and SNAP-25 coupled Luminex beads where in this case only SNAP-25 is cleaved at the BoNT/A-specific cleavage site. After adding a mixture of Neo-mAbs specific for BoNT/A and BoNT/B, only BoNT/A-specific Neo-mAbs bind to cleaved SNAP-25 immobilised to the colour-coded Luminex-beads enabling specific discrimination between one SNAP-25 and one VAMP-2 cleaving serotype in one assay. The fluorescence-intensity correlates with the amount of cleaved substrate and therefore indicates the concentration of BoNT in the sample.

As an alternative, we swapped orientation of the assay set-up and immobilised SNAP-25 and VAMP-2 on two different Luminex bead regions. By adding the immuno-enriched toxins directly to the Luminex beads with immobilised substrates, an on-bead cleavage allows for the discrimination between one SNAP-25- and one VAMP-2-specific BoNT serotype at a time (Fig. 3). Here, the experimental conditions were optimised to ensure maximum sensitivities by identifying a cleavage buffer that allowed for efficient cleavage of all serotypes. In addition, we evaluated different cleavage durations (30 min to 18 h) achieving maximum sensitivity after overnight incubation (Fig. S5). After cleavage, the newly formed neoepitopes were detected by Neo-mAbs. By combining Neo-mAbs against one SNAP-25- and one VAMP-2-cleaving serotype, three duplex-assays for the simultaneous detection of either serotypes BoNT/A and B or BoNT/E and F (all pathogenic to humans) as well as BoNT/C and D as solely veterinarian relevant serotypes were established (Fig. 3).

#### Sensitivity for detection of purified BoNT after enrichment from buffer

**Figure 4:**
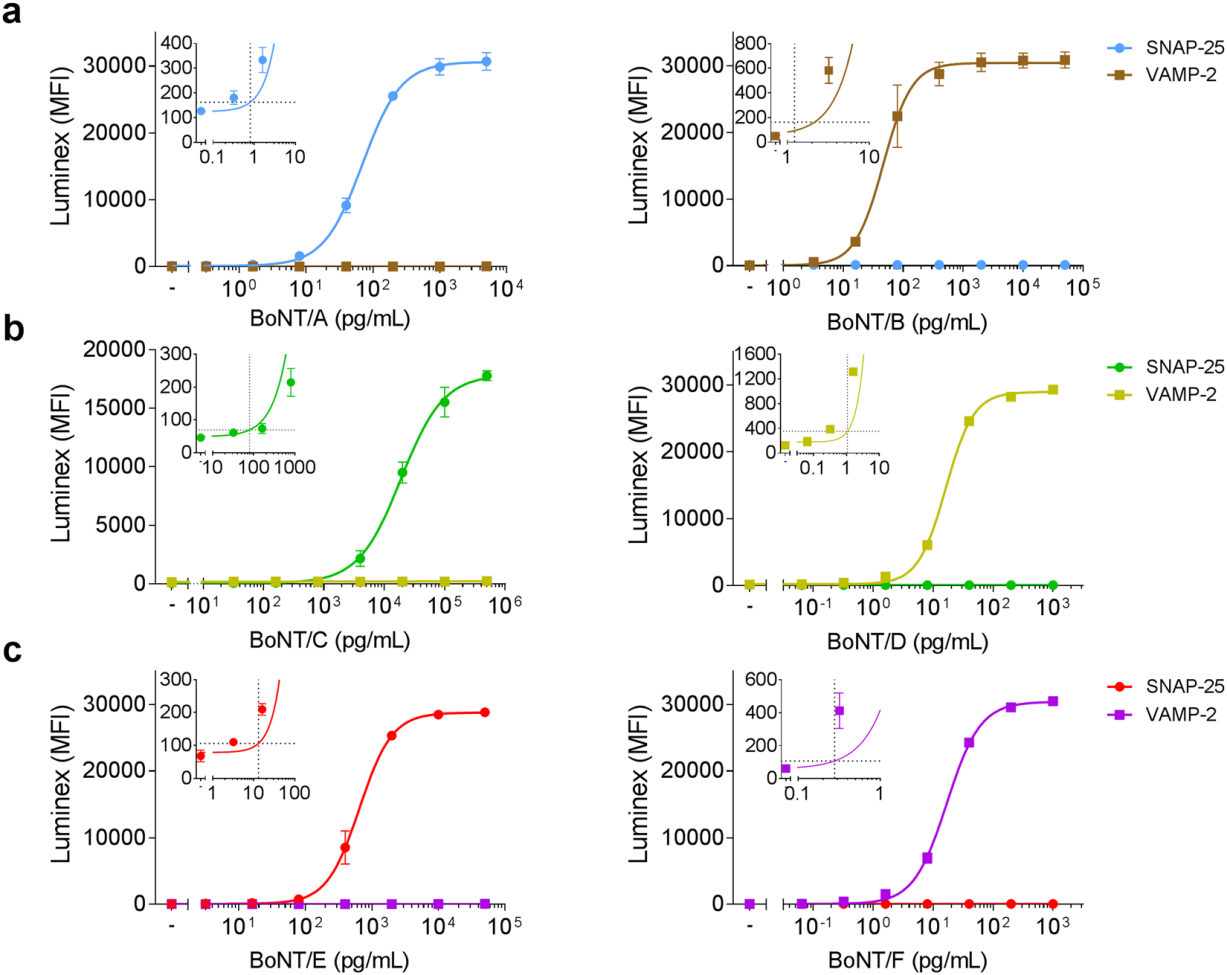
Sensitivity and specificity of BoNT detection by three Luminex duplex-assays. As indicated in Fig. 3, the different serotypes BoNT/A to F were diluted in BSA/PBS and enriched with monoclonal anti-BoNT antibodies coupled to paramagnetic beads (duplex enrichment: A + B, C + D, E + F). Captured toxin was mixed with VAMP-2 and SNAP-25 coupled to Luminex microspheres for substrate cleavage and cleavage products were subsequently detected by a mixture of Neo-mAbs targeting the cleavage sites of BoNT/A and B (**a**), BoNT/C and D (**b**), or BoNT/E and F (**c**). Results from two independent experiments with each repeat performed in technical duplicates are shown. (n = 4; mean ± SD; MFI = Median fluorescent intensity). Inserts show cut-off values (horizontal dashed lines) used to calculate the limits of detection (vertical dashed lines) zoomed in on the relevant areas of the graphs.

To evaluate the assay sensitivity and to exclude cross-reactivity between the SNAP-25 and VAMP-2 coated Luminex beads, the different BoNT serotypes were titrated and detected with the respective duplex-assays (Fig. 4 and Table 2). The three duplex-assays were highly specific for the respective BoNT added since no cross-reactivity between the SNAP-25- and the VAMP-2-cleaving serotype tested simultaneously in one assay was observed. Sensitivity of the two duplex-assays for BoNTs pathogenic to humans (A + B, E + F) was excellent and ranged between 0.3–13 pg/mL whereas the veterinary relevant serotypes BoNT/C and D were detected with detection limits of 79.1 pg/mL and 1.1 pg/mL, respectively, which is well in the range of the MBA (Table 2).

**Table 2:** Assay performance of the duplex-assay for detection of BoNT/A-F substrate cleavage.

	BoNT/A	BoNT/B	BoNT/C	BoNT/D	BoNT/E	BoNT/F
EC50 (pg/mL)	70	47	18901	16	654^c^	17
LOD (pg/mL)	0.8	1.2	79.1	1.1	13	0.3
(95% CI)^a^	(0.6–1.3)	(0.7–5.4)	(45–227)	(0.9–1.3)	(9.2–23.7)	(0.2–0.4)
LOD (LD_50_/mL)^b^	0.211	0.147	2.056	0.18^d^	0.84	0.005

^a^Cutoff: mean + 3.29 × SD of blank values (determined with 36 blank values for each serotype) including lower and upper limit of 95% confidence interval (CI; shown in parentheses). ^b^Limit of detection (LOD) in mouse minimal lethal doses (LD_50_/mL) according to toxin activity as specified by the manufacturer (see Methods). ^c^trypsinated BoNT/E. ^d^Calculated according to Sugiyama^35^.

To elucidate the robustness of the duplex-assays we performed an initial interlaboratory comparison study exemplarily for the BoNT/A + B duplex-assay. Here, excellent agreement of results between the two laboratories was found, showing that the duplex-assay is robust and yields reproducible results independent of the laboratory or experimenter, a finding that will have to be corroborated in the future more extensively in larger interlaboratory exercises (Fig. S6).

#### Detection from complex matrices

Finally, to evaluate whether the three duplex-assays are applicable for the functional detection of BoNTs in complex matrices, we tested detection in spiked serum samples as the most common clinical sample matrix. Furthermore, detection of the most prevalent serotypes BoNT/A and B from different food matrices was evaluated. To cover a broad range of different toxin concentrations, we spiked toxin concentrations near the EC_50_ value (medium toxin concentration), in the lower (low toxin concentration, EC_50_/10) and upper (high toxin concentration, EC_50_ × 10) region of the titration curve for each serotype and determined recovery rates from spiked serum or food samples compared to spiked BSA/PBS (Table 3 and Figs S7 and S8).

**Table 3:** Recovery (%) of BoNT from complex matrices at low, medium or high concentration range in the Luminex duplex-assays.

Matrix	Toxin	Low^a,b^	Medium^b^	High^b^
Serum	BoNT/A	58 ± 25.8	85 ± 13.4	99 ± 1.8
	BoNT/B	45 ± 3.1	58 ± 10.7	92 ± 1.7
	BoNT/C	156 ± 30	100 ± 7.9	98 ± 3.7
	BoNT/D	89 ± 11.9	89 ± 7.1	98 ± 1.9
	BoNT/E	40 ± 5.0	34 ± 7.2	96 ± 1.5
	BoNT/F	83 ± 4.2	81 ± 7.0	99 ± 2.7
Fish	BoNT/A	76 ± 13.5	93 ± 29	100 ± 1.8
	BoNT/B	n.d.^c^	11 ± 8.8	11 ± 1.7
Sausage	BoNT/A	62 ± 21.4	51 ± 6.9	96 ± 5
	BoNT/B	78 ± 25.6	48 ± 21.9	74 ± 22.9
Beans	BoNT/A	113 ± 32.2	95 ± 18.8	97 ± 3.4
	BoNT/B	72 ± 31	43 ± 14.8	85 ± 12.5

^a^Spiked low toxin concentrations in serum for BoNT/A to F: 7 (A), 5 (B), 2000 (C), 2 (D), 70 (E), 2 (F) pg/mL. Medium toxin concentrations were near the EC_50_ value, low toxin concentration: EC_50_/10 and high toxin concentration: EC_50_ × 10. For detection from food matrices, the low concentration was adjusted to 15 pg/mL for BoNT/A and 5 pg/mL for BoNT/B. ^b^Toxin recovery from spiked serum samples compared to spiked BSA/PBS as reference (100%) was calculated. Results from two independent experiments, each performed in technical duplicates (n = 4; mean ± SD). ^c^n.d. = not detectable.

For detection from serum, all toxin concentrations tested were recovered successfully. As expected, excellent recovery rates of nearly 100% were achieved when high toxin concentration were spiked. At medium toxin concentration almost complete recovery could be achieved for BoNT/A, C, D, and F (81–100%) while slightly reduced recovery rates were observed for BoNT/B and E correlating with the lower affinities of the monoclonal antibodies used for enrichment (Table S1). Importantly, even at the lowest concentrations measured (7 pg/mL for BoNT/A, 5 pg/mL for BoNT/B, 2 ng/mL for BoNT/C, 2 pg/mL for BoNT/D, 70 pg/mL for BoNT/E and 2 pg/mL for BoNT/F) recovery rates of at least 40% indicated that the duplex assay could be employed successfully for the detection of all six BoNTs from serum in a diagnostic setting (Table 3 and Fig. S7). This is an important finding, since serum is the clinical matrix most frequently tested by MBA in reference laboratories in Europe and beyond during the course of an outbreak investigation.

In the case of three food matrices tested so far, only a slight or no impact on detection of BoNT/A and B was observed (Table 3 and Fig. S8). Here, BoNT/A was detected from all three matrices at recovery rates of at least 50%. Similarly, BoNT/B was recovered from all food matrices at recovery rates >43% except for fish, which negatively influenced detection. Here, the lowest spiked toxin concentration (5 pg/mL BoNT/B) could not be detected (Fig. S8). However, medium and high concentrations could still be detected reliably also for BoNT/B showing that detection from food matrices linked to food botulism was possible for both BoNT/A and BoNT/B using the duplex assay. Future work will have to extend the assay validation with a broader set of different food matrices.

## Discussion

In this work, we established a novel functional suspension array for the detection of all “classical” clinically relevant and catalytically active BoNT serotypes A through F based on the Luminex platform. The assay is highly sensitive – in the range of the MBA or significantly better – and applicable to clinical and food matrices. As an asset of the novel approach, a comprehensive panel of Neo-mAbs specifically recognising the individual cleavage products of the synaptic substrates SNAP-25 or VAMP-2 after proteolysis by BoNT/A to BoNT/F was generated and characterised.

The development of replacement methods for the MBA for botulism diagnostics is highly desirable due to the distress inflicted on the experimental animals. In light of the current legislation in the European Union, the effective Directive 2010/63/EU of the European Parliament on the protection of animals used for scientific purposes stipulates in Article 4 the strict adherence to the Russel’s and Burch’s 3 R principles, reduction, refinement and replacement of animals, wherever possible^69,70^. However, implementing the 3 R principles for botulism diagnostics is highly challenging due to the molecular variability of pathogenic BoNT serotypes covering several immunologically distinct serotypes with more than 40 subtypes and mosaics^31,32^. The analytical challenge is to comprehensively detect all relevant sero- and subtypes even from complex matrices such as serum or food without risking false negatives. Due to the coverage of the complete mode of action of all mouse-pathogenic sero- and subtypes, the MBA is still seen as the gold standard assay for botulism diagnostics, despite of its technical and ethical limitations^31,33^.

The disadvantages associated with the MBA have driven the development of *in vitro* methods displaying either the abundance of the protein (*e.g*. ELISA- or MS-based methods), the presence of the toxin genes (PCR-based assays) or assays depicting partial or full BoNT activity (*e.g*. Mouse Phrenic Nerve Hemidiaphragma Assay, Endopep-MS or cell-based assays^31,32,71^). Still, there is currently no widely accepted, straightforward animal replacement method available to routine laboratories for botulism diagnostics. A recent first international proficiency test to evaluate existing BoNT detection methods by comparing *in vitro* methods with the MBA showed that among several methods run in parallel on the same sample set, Endopep-MS and Endopep-ELISA approaches, which both do not require animals or animal tissues, delivered qualitative and/or quantitative results similar to or better than the MBA^59,72^. Among those, the applicability of the Endopep-MS assay is somewhat limited due to the high level of expertise and expensive technical instrumentation needed^73^. On the other hand, assays with a broader applicability based on fluorescence or luminescence read-outs after cleavage of modified peptides usually lack serotype-specificity and/or sensitivity, although an extremely sensitive assay for BoNT/A has been reported^45^. Addressing the current limitations, the starting point for this work was the idea that an Endopep-ELISA based assay using Neo-mAbs would combine the advantage of being applicable in routine labs using commonly available instrumentation with high sensitivity and specificity, provided that appropriate Neo-mAbs of high specificity would become available.

Along this line, a major achievement of this work was to generate and characterise a comprehensive panel of 20 Neo-mAbs specifically detecting the cleavage products of the substrates of BoNT/A, B, C, D, E and F, with six outperforming Neo-mAbs selected for further assay development. BoNT/G was omitted at this stage as no natural cases of botulism caused by this serotype have been reported so far. One notable characteristic of the Neo-mAbs is the exquisite specificity for their respective cleavage product with no cross-reactivity to the cleavage products of other serotypes. This high specificity was especially remarkable for the Neo-mAbs targeting the SNAP-25 cleavage products of BoNT/A and C or VAMP-2 cleavage products of BoNT/D and F, where the individual cleavage sites are separated by only one terminal amino acid position. Until then, a similar specificity has only been observed for polyclonal antibodies targeting BoNT/A or C cleaved SNAP-25^54,55^. The observed high specificity, together with the lack of cross-reactivity against intact substrates, indicates a common mode of binding of the Neo-mAbs: the binding seems to be based on burying the newly exposed termini in deep binding pockets with critical contribution of the terminal amino acids, a feature that has recently been demonstrated for Bapineuzumab, a Neo-mAb targeting the N-terminal epitope of amyloid-beta peptide in Alzheimer disease^74^.

In order to detect the catalytic activity of different BoNT serotypes, the precise identification of each cleavage product by Neo-mAbs has major advantages compared to other detection methods solely analysing substrate cleavage. FRET based assays, for example, do not allow distinguishing between serotypes targeting the same substrate. In addition, those assays bear a higher risk of giving false positive results, as unspecific substrate cleavage induced by matrix proteases would result in false positive signals. Assays employing Neo-mAbs, however, only give positive signals, if the respective BoNT substrate is cleaved at the exact serotype specific position. Here, false positive results could also occur due to the ability of trypsin to cleave at the cleavage sites for BoNT serotypes C, D, and E, yet at a lower frequency compared to FRET substrates. Additionally, false positive results would be identified in the multiplexed approach as simultaneous positive signals for the abovementioned serotypes are indicative of a contamination with trypsin.

The six selected Neo-mAbs targeting the substrate cleavage site of BoNT/A to F were implemented in a set of three duplex-assays based on the Luminex platform simultaneously detecting the catalytic activity of two different serotypes (BoNT/A + B, C + D, or E + F). In fact, our suspension array represents the first implementation of an endopeptidase assay on the Luminex platform. To date, only few functional Luminex assays have been published displaying *e.g*. tyrosine kinase activation by using phospho-tyrosine-specific antibodies^75,76^. Hence, the Luminex platform turns out to be instrumental to display abundance, expression pattern, and activation status of target molecules.

The novel functional suspension array represents a major advancement in BoNT diagnostics since all serotypes pathogenic to humans can be tested in two parallel tests combining the A + B and the E + F duplex assays using minimal sample volumes (50–200 µL). In case of veterinary botulism, the duplex assay detecting the enzymatic activity of BoNT/C + D complements the set of ELISA and MS-based detection methods previously established in our laboratory^77^. The overall sensitivity of the three duplex-assays was in the range or even higher than the MBA, is comparable to Endopep-MS based cleavage assays^41^, and approximately 10-times more sensitive compared to FRET-or luminescence based assays^47,48,78^. Furthermore, the functional suspension array reaches this sensitivity in an assay time of less than 24 h, as compared to the MBA which can take up to 4 days. More sensitive assays have also been described based on either surface plasmon resonance measurements or Endopep-ELISA for BoNT/A, BoNT/B and BoNT/E with limits of detections as low as 0.01 LD50^55,61,62,79,80^. Those higher sensitivities could in part be explained by omitting enrichment steps necessary for broad detection from complex matrices. Another factor could be the possibility to optimise the assay conditions regarding cleavage buffer for selected, individual serotypes whereas our assay was optimised for detection of all six serotypes. However, the excellent sensitivities obtained in the SPR based assays also indicate that assay specific advantages in conjunction with excellent cleavage specific mAbs might contribute to the superior sensitivity observed in those assays.

The Neo-mAbs generated in this work might also be useful tools to extend alternative detection platforms, *e.g*. surface plasmon resonance sensors based on poly- or monoclonal Neo-Abs binding cleaved SNAP-25 or VAMP-2 for detection of BoNT/A, B, or E^62,63,79,81,82^. In fact, although in this work we tested detection of cleaved VAMP-2 only, our antibodies should also bind to cleaved VAMP-1 and -3 as the recognition site is conserved across the three isoforms hereby further broadening the applicability our mAbs. Although the focus of this work was clearly on the detection of BoNTs in a diagnostic setting, the Neo-mAbs could also be useful for the development of assays replacing the MBA in potency testing of highly pure pharmaceutical BoNT preparations. Here, an FDA approved assay for the determination of the specific activity of BoNT/A relies on combining a cell based assay with a cleavage-based readout by a Neo-mAb specific for the cleavage site for BoNT/A^60^. In the same context, a recent bifunctional assay detecting both receptor binding and substrate cleavage based on a polyclonal Neo-Ab has been proposed for potency testing of BoNT/A and BoNT/B^57,83^. Here, possible lot to lot variation by polyclonal Neo-Abs could be avoided by inclusion of Neo-mAbs. Additionally, as both tetanus toxin (TeNT) and BoNT/B share the same cleavage site on VAMP, our neoepitope antibodies detecting BoNT/B cleavage should be applicable for assays detecting enzymatically active TeNT^84^.

Similarly, in a diagnostic setting endogenous receptor binding could be an alternative for antibody extraction of toxin from complex matrices. This principle has been explored for selected serotypes by usage of recombinant receptor molecules, receptor fragments^57,83,85,86^, or synaptosomes for BoNT/A, B, or F^87–90^. However, the implementation of the BoNT receptor interaction of all serotypes *in vitro* is highly challenging due to the molecular structure of the BoNT receptors. Recent evidence extended the long-standing two-receptor binding paradigm, the binding of BoNTs to a specific transmembrane protein receptor and a ganglioside^25^. Additionally to the known receptors, BoNT/A does need a highly specific post-translational modification, a glycosylation, on the protein receptor to confer high-affinity binding^85,91^. Likewise, BoNT/B, DC, and G were shown to employ ternary interactions by binding to a protein, ganglioside, and membrane lipids^92,93^. In the future, innovative approaches are necessary to display all components of the endogenous receptors including any post-translational modification *in vitro*. In a diagnostic setting, only the successful implementation of high-affinity binding would result in detection limits necessary to replace the MBA.

Very recently, several novel BoNT- and BoNT-like molecules have been identified which all target novel cleavage sites in different synaptic substrates^10,11,13–15^. The relevance of those novel BoNT-like molecules for human disease is currently under investigation. BoNT/HA which targets the same cleavage site in VAMP-2 as BoNT/F5 has been identified from a bivalent strain co-expressing BoNT/B in a single case of infant botulism^10,11^. BoNT/X targets a novel site in VAMP-1,2,3 and several non-canonical substrates and is also present in a bivalent BoNT/B expressing strain isolated from a single infant botulism case. The LH_N_ of BoNT/X cleaves VAMP-2 and VAMP-4 in cultured neurons and a sortase ligated BoNT/X induces flaccid paralysis in mice at µg dosage, so it could naturally be associated with neurological diseases, although this has not been demonstrated so far^13^. LC of eBoNT/J has been shown to cleave both SNAP-25 and VAMP-2 *in vitro* at sites distinct from known BoNT cleavage sites, but the host species targeted and any potential association with disease remains to be determined^14^. As soon as a link to botulism – or any other human condition – is established, the development of Neo-mAbs targeting the specific cleavage site(s) could be addressed transferring the current multiplex endopeptidase approach to the novel BoNT- and BoNT-like molecules. Along this line, work on Neo-mAbs specific for the BoNT/HA / BoNT/F5 cleavage site is ongoing in our laboratory. In a broader context, our work exemplifies how specific Neo-mAbs against enzymatic cleavage sites can be used in multiplexed assays to diagnose human disease – an approach that can be transferred to other human conditions involving small epitope changes in biomolecules.

In conclusion, the functional suspension array for the detection of all clinically relevant BoNT serotypes A through F based on the Luminex platform in conjunction with the unique panel of Neo-mAbs represent a significant advancement towards the replacement of the mouse bioassay for botulism diagnostics. Both the assay and the Neo-mAbs are now available to routine laboratories upon request strengthening their diagnostic capabilities and significantly reducing the number of animals needed for botulism diagnostics.

## Methods

### Chemicals and toxins

The following chemicals were used in the experiments: Coomassie brilliant blue G250 (Bio-Rad, Munich, Germany), Trimethylamine N-oxide dihydrate (TMAO) (Sigma-Aldrich, Munich, Germany). All other chemicals and reagents were obtained from Carl Roth, Sigma-Aldrich or Merck. Botulinum neurotoxin serotypes A1 (2.6 × 10^8^ mouse LD_50_/mg), B1 (1.2 × 10^8^ mouse LD_50_/mg), C (Endopep-ELISA: 3 × 10^7^ mouse LD_50_/mg; Western Blots and duplex-assay: 2.6 × 10^7^ LD_50_/mg), D (9.6 × 10^7^ mouse LD_50_/mg; identified as BoNT/DC^94^, with BoNT/D and DC using the same cleavage site in VAMP-2), E3 (non-trypsinised: 3 × 10^5^ mouse LD_50_/mg; trypsinised: 6 × 10^7^ mouse LD_50_/mg), and F1 (1.8 × 10^7^ mouse LD_50_/mg) were obtained from Metabiologics (Wisconsin, USA) as proteins purified from *C. botulinum* supernatants. Recombinant BoNT/D for duplex-assay experiments was obtained from Toxogen (Hannover, Germany). For duplex-assay experiments trypsin activated BoNT/E was used. Trypsin activation was performed prior to the experiments using Mag-Trypsin (TPCK-trypsin immobilised on magnetic beads; Takara Bio, Heidelberg, Germany) according to the manufacturer’s instructions. Toxins were always handled in a biosafety cabinet. For inactivation, liquid waste was decontaminated by incubation with 5% NaOH for at least 24 h. Consumables that were in contact with toxin containing solutions were flushed with 5% NaOH and autoclaved (60 min at 134 °C).

### Expression and purification of full-length rSNAP-25H6 and H6trVAMP-2

Recombinant rat SNAP-25 amino acid 1–206 fused to a C-terminal His6tag (rSNAP-25H6) was expressed in *E. coli* M15 strain as described previously^95^. The plasmid pET15b-VAMP-2 encoding rat VAMP-2 amino acid 1–97 fused to an N-terminal thrombin cleavable His6tag (H6trVAMP-2 1–97) was expressed in *E. coli* BL21DE3 strain upon induction by IPTG 55. *E. coli* cells were harvested, resuspended in 50 mM Tris-HCl, pH 8.0, 150 mM NaCl, 5 mM imidazole and protease inhibitor EDTA-free Complete (Roche, Penzberg, Germany) and lysed by ultrasound. rSNAP-25H6 and H6trVAMP-2 1–97 were isolated by IMAC using Talon matrix (Takara Bio, Heidelberg, Germany), washed in resuspension buffer supplemented with 1 M NaCl and eluted in resuspension buffer supplemented with 250 mM imidazole. The proteins were polished by gel filtration (Superdex-75, GE Healthcare, Freiburg, Germany), rSNAP-25H6 in 20 mM HEPES-KOH, pH 7.4, 150 mM KCl and H6trVAMP-2 1–97 in PBS, pH 7.4. Desired fractions containing the recombinant proteins were pooled, frozen in liquid nitrogen and kept at −70 °C. Protein concentrations were determined subsequent to 15% SDS-PAGE and Coomassie blue staining by using a LAS-3000 imaging system (FUJIFILM Europe GmbH, Düsseldorf, Germany), the AIDA 3.51 software (Raytest, Berlin, Germany) and BSA (100–1600 ng) as reference protein.

### Generation of monoclonal antibodies

Immunisation and euthanisation of mice for the production of monoclonal antibodies was performed in agreement with the European legislation for the protection of animals used for scientific purposes (Directive 2010/63/EU). Approval of experiments was given by the State Office for Health and Social Affairs in Berlin (LaGeSo Berlin, Germany) under the registration number H0109/03. Monoclonal mouse antibodies used for toxin enrichment were if not published earlier^77^ raised against recombinant H_C_ fragments (Toxogen, Hannover, Germany) of BoNT/A, BoNT/B, or BoNT/F and generated as described previously^58,96^. For generating neoepitope specific antibodies, mice (BALB/c or NMRI) bred under pathogen-free conditions at Charles River (Sulzfeld, Germany) were used for immunisations starting at the age of eight weeks. For immunisation, BSA-coupled peptides corresponding to the respective cleavage site of each BoNT molecule were used (BoNT/A: BSA-CTRIDEANQ; BoNT/B: BSA-CALQAGASQ; FETSAAKLC-BSA; BoNT/C: BSA-CRIDEANQR, ATKMLGSGC-BSA; BoNT/D: LSELDDRAC-BSA; BoNT/E: BSA-CTQNRQIDR, IMEKADSN-BSA; BoNT/F: BSA-CVDKLERDQ, KLSELDDRC-BSA; Petra Henklein, Institute for Biochemistry, Charité Berlin). Per immunisation 100 µg BSA-coupled peptides (one single peptide or a mix of up to six different peptides) in complete (first immunisation) or incomplete (booster immunisations) Freund’s adjuvant (Sigma-Aldrich, München, Germany) were injected intraperitoneally into mice in four-week intervals until a sufficiently high antibody titre was obtained. Final boosts were performed on days –3, –2, and –1 before fusion with the same dose of antigens diluted in PBS. On days 10 to 19 post-fusion, specificity of hybridoma clones was tested by indirect ELISA using the corresponding KLH-coupled peptide (see below). Clones were subcloned at least twice. IgG-antibodies were purified by affinity chromatography using a HiTrap MabSelect SuRe column on an Äkta Protein Purification System (GE Healthcare Bio-Sciences AB, Uppsala, Sweden) and subsequently dialysed against PBS for storage at 4 °C (short term storage) or –80 °C (long term storage), respectively. For implementation in the Luminex duplex assay, Neo-mAbs were biotinylated and subsequently desalted using Biotinamidohexanoyl-6-aminohexanoic acid N-hydroxysuccinimide ester (Sigma Aldrich, Munich, Germany) at a molar ratio of 20 according to manufacturer’s recommendations. Protein concentrations were determined spectrometrically at an Implen Nanophotometer (Munich, Germany) using IgG extinction coefficients and biotinylated mAbs were stored in PBS containing 0.05% (w/v) sodium azide and a final concentration of 0.2% (w/v) BSA.

### Indirect enzyme-linked immunosorbent assay (ELISA)

To determine specificity towards the respective BoNT cleavage site and to exclude cross-reactivity towards the full-length substrates, hybridoma clones and purified Neo-mAbs were analysed by indirect ELISA using the following antigens: KLH-coupled peptides corresponding to the respective cleavage site of each BoNT molecule were used (sequences analogue to the BSA-coupled peptides; obtained from Petra Henklein, Institute for Biochemistry, Charité, Berlin), KLH (Sigma-Aldrich, Munich, Germany), BSA, H6trVAMP-2, and rSNAP-25H6. For the indirect ELISA 50 µL antigen diluted to 0.5 µg/mL in 1 µg/mL BSA/PBS were coated on MaxiSorp microtitre plates (F96; Nunc, Thermo Fisher Scientific, Langenselbold, Germany) overnight at 4 °C. Plates were washed (4×) with 300 µL PBS-T (PBS supplemented with 0.1% Tween 20) and blocked by adding 200 µL per well 2% skimmed milk powder in PBS-T for 1 h at room temperature. After washing, 50 µL hybridoma supernatants or purified Neo-mAbs (10 µg/mL) were added and incubated for 1 h before plates were washed again with and incubated with 50 µL per well horseradish peroxidase (HRP) goat-anti-mouse IgG (Fcγ) specific antibody (1:2500; Dianova, Hamburg, Germany) for 30 min. Finally, plates were washed 8× and developed using 100 µL per well 3,3′,5,5′-tetramethylbenzidine (TMB, SeramunBlau slow; Seramun, Heidesee, Germany) for 15 minutes and stopped by adding 100 µL per well 0.25 M H_2_SO_4_. Absorption was read at 420 nm referenced to 620 nm by an ELISA reader (Tecan; Crailsheim, Germany).

### SDS-PAGE and Western Blots

Antibodies were tested for their exclusive specificity towards the cleaved but not the intact substrate using Western blotting. To this end, 6 µM H6trVAMP-2 or rSNAP-25H6 were incubated with 200 nM BoNT/A, B, E or F, 40 nM BoNT/D, or 500 nM BoNT/C in cleavage buffer (50 mM HEPES, 250 µM ZnCl_2_, 1% Tween 20, 0.75 M TMAO, 25 mM DTT, pH 7) for 18 h at 37 °C under constant agitation in a thermomixer at 600 rpm (Eppendorf, Hamburg, Germany). Samples were inactivated by heating to 95 °C for 10 minutes. Subsequently, samples were supplemented with 3 × Laemmli loading buffer (150 mM Tris/HCl pH 6.8, 6% SDS, 30% glycerol, 7.5% β-mercaptoethanol, 0.25% bromophenol blue) at a 1:3 ratio, heated for 10 minutes at 70 °C and cooled down to 4 °C. For electrophoretic protein separation, samples were loaded on 4–20% mini-PROTEAN® TGX™ precast protein gels (Bio-Rad, Munich, Germany; for mAb VAMP/D/27 only) or on 16% polyacrylamide gels^97^. Gels were then stained with colloidal Coomassie blue^98^ or applied for Western blotting. For the latter, gels were blotted onto an Immuno-Blot 0.2 µm (VAMP/D/27 only) or 0.45 μm PVDF membrane (Thermo Fisher Scientific, Langenselbold, Germany), blocked in blocking buffer (2% skimmed milk in PBS-T) and incubated overnight at 4 °C (or 1 h at RT for mAb VAMP/D/27) with 10 µg/mL neoepitope specific antibody diluted in blocking buffer. After three washing steps with blocking buffer, membranes were incubated with a horseradish peroxidase (HRP)-coupled goat anti-mouse IgG Fc antibody (Dianova, Hamburg, Germany) diluted in blocking buffer (1:2500) for 30 minutes (or 1 h for mAb VAMP/D/27) at room temperature, washed three times with PBS-T, and developed using SuperSignalWest Dura Extended Duration Substrate (Thermo Fisher Scientific, Langenselbold, Germany). All images were documented at a ChemiDoc workstation (Bio-Rad, Munich, Germany).

### Endopeptidase-ELISA

The endopeptidase-ELISA was performed according to Jones *et al*.^55^ using a slightly modified protocol. 3 µg/mL of rSNAP-25H6 or H6trVAMP-2 diluted in 50 mM carbonate-buffer (pH 9.6) were coated on MaxiSorp microtitre plates overnight at 4 °C. On the next day, coating solution was decanted and plates were blocked with 5% skimmed milk powder PBS-T for 90 minutes at room temperature. Subsequently, plates were washed with ddH_2_O (4 × ), dried, and toxin diluted in cleavage buffer (see above) was added. Plates were incubated for 18 h at 37 °C with constant back and forth movement in a hybridisation oven (Biometra GmbH, Göttingen, Germany) for substrate cleavage. Plates were then washed with PBS-T (4×) and incubated with 100 µL neoepitope-specific antibodies diluted to 1 µg/mL in 2% skimmed milk powder PBS-T for 90 minutes at room temperature on a multiwell plate shaker (IKA, Staufen, Germany) at 300 rpm for the detection of cleavage products. After incubation with Neo-mAbs, plates were washed again with PBS-T (4×) and incubated with 100 µL HRP-coupled goat anti-mouse IgG H + L (1:2500 dilution, Dianova, Hamburg, Germany) secondary antibody diluted in 2% skimmed milk powder PBS-T for 30 minutes at room temperature on a multiwell plate shaker at 300 rpm. Finally, plates were washed with PBS-T (8×) and developed as described above.

### Luminex duplex-assays

For toxin enrichment, 150 µg antibodies specific for BoNT/A to F (Supporting Table 1) were covalently coupled to 250 µL Dynabeads M-270 carboxylic acid (Thermo Fisher Scientific, Langenselbold, Germany) according to the manufacturer’s instruction. 200 µL toxin solution diluted either in 0.1%BSA/PBS or serum (pooled from 8 human individuals, not inactivated) or homogenised foods diluted 1:10 with 0.1%BSA/PBS were incubated with 10 µL beads for 1 h at room temperature on a multiwell plate shaker at 600 rpm. Beads were then washed with PBS-T (2×) and ddH_2_O (2×). After washing, beads were resuspended in 100 µL cleavage buffer (see above) and applied in the duplex-assay. To detect two distinct BoNT serotypes in one sample, the Bio-Plex^®^ Multiplex Immunoassay System (Bio-Rad, Munich, Germany) was used. BoNT substrates SNAP-25 or VAMP-2 were coupled to 1.5 × 10^6^ beads (10 µg substrate, region 008 or 048, respectively) of MagPlex^®^ Microspheres (Luminex Corporation, Austin, TX, USA) according to the manufacturer’s instructions. For BoNT/A + B and BoNT/E + F duplex-assays, the substrates SNAP-25 (ATGen Co. Ltd., Seongnam-si, Korea) and VAMP-2 (ProSpec-Tany TechnoGene Ltd., Ness Ziona, Israel) were used. For BoNT/C + D duplex-assay rSNAP-25H6 and H6trVAMP-2 (see above) were used. For substrate cleavage (on beads) toxins bound to enrichment beads resuspended in cleavage buffer were mixed with SNAP-25 and VAMP-2 microspheres (2500 microspheres/100 µL sample) in microtitre plates (96 well Microplate, Greiner Bio One, Frickenhausen, Germany) and incubated for 18 h at 37 °C under constant back and forth movement in a hybridisation oven. Microspheres were then washed with PBS-T (2×) and incubated with biotinylated Neo-mAbs SNAP/A/291 + VAMP/B/1148 (1:100 and 1:75), SNAP/C/5593 + VAMP/D/27 (1:7500 and 1:2500), or SNAP/E/217 + VAMP/F/425 (1:1000 and 1:500) diluted in assay buffer (1% BSA/PBS) for 90 minutes on a multiwell plate shaker at 600 rpm. Microspheres were washed with PBS-T (2×) and incubated with 2 µg/mL streptavidin-R-phycoerythrin (PhycoLink Streptavidin-R-Phycoerythrin PJRS34; ProZyme, Hayward, USA) diluted in assay buffer for 30 minutes on a multiwell plate shaker at 600 rpm. Finally, microspheres were washed with PBS-T (3×), resuspended in assay buffer and analysed using the Bio-Plex 200 system (Bio-Rad, Munich, Germany).

## Supplementary information

Supplementary Information

## Data Availability

All data generated or analysed during this study are included in this published article and its Supplementary Information files.
