# Supplementary material for: Functional detection of botulinum neurotoxin serotypes A to F by monoclonal neoepitope-specific antibodies and suspension array technology
Source: Sci Rep. 2019 Apr 2;9:5531. doi: 10.1038/s41598-019-41722-z (PMC6445094; doi:10.1038/s41598-019-41722-z)
Supplement: Supplementary file 1 — Supplementary Information [file 41598_2019_41722_MOESM1_ESM.docx]

Supplementary Information

Functional detection of botulinum neurotoxin serotypes A to F by monoclonal neoepitope-specific antibodies and suspension array technology

**Laura von Berg^1^, Daniel Stern^1^, Diana Pauly^1,4^, Stefan Mahrhold^2^, Jasmin Weisemann^2^, Lisa Jentsch^1^, Eva-Maria Hansbauer^1^, Christian Müller^3^, Marc A. Avondet^3^, Andreas Rummel^2^, Martin B. Dorner^1^, and Brigitte G. Dorner^1,^***

# Supporting methods

## Surface plasmon resonance measurements (Biacore)

Antibody affinity and kinetics of monoclonal anti-BoNT antibodies used for enrichment was analysed by surface plasmon resonance (SPR) technology using a Biacore T200 instrument (GE Healthcare, Freiburg, Germany) at 25 °C. For all measurements antibodies and analytes were diluted in HBS-EP+ running buffer (10 mM HEPES, 150 mM NaCl, 3 mM EDTA, 0.05% Tween-20, pH 7.4).

To this aim, monoclonal antibodies were immobilised using an anti-mouse antibody capture kit (GE Healthcare, Freiburg, Germany) modified series S CM5 sensor chip by injecting 50 µg/mL antibody for 120 s at 5 µL/min on flow cell 2 (Fc2) leading to immobilisation levels between 50 and 150 resonance units (RUs) of captured antibodies. Fc1 remained empty (anti-mouse capture antibody only) and was set as blank control. Binding kinetics were determined by injecting serial dilutions (1:3 dilutions) of 50 kDa BoNT H_C_ fragments (A2807: H_C_A1 5 µg/mL to 0.0617 µg/mL; B488: H_C_B1 30 µg/mL to 0.37 µg/mL; C9: H_C_C 30 µg/mL to 0.37037 µg/mL; D967: H_C_D 5 µg/mL to 0.0617 µg/mL; F1726: H_C_F1 1 µg/mL to 0.0123 µg/mL) or full-length BoNT/E3 (150 kDa; 90 to 1.11 µg/mL; Metabiologics, Madison, WI, USA) over Fc1 and Fc2 for 120 s at 30 µL/min followed by a 600 s injection of running buffer. For sensor chip regeneration, 10 mM Glycin pH 1.7 (GE- Healthcare) was injected for 180 s at 10 µL/min. To ensure reproducibility of measurements and stable sensor surface activity the highest analyte concentration was measured in duplicates. In addition, duplicate blank measurements (no analyte) were performed in each run.

Data was analysed using the Biacore T200 Evaluation Software (3.01; GE Healthcare). To this aim, double referenced ^1^ sensorgrams were fit to the 1:1 Langmuir binding model (A + B = AB) setting R_max_ local to allow for adaption to slightly different immobilisation levels caused by the use of the capture kit.

## Multiplex Luminex assay

For substrate cleavage, a mixture of 8 µg/mL biotinylated SNAP-25 (aa137-206) and 25 ng/mL biotinylated VAMP-2 (aa33-94) in the singleplex assays or a mixture of 8 µg/mL biotinylated SNAP-25 (aa137-206), 25 ng/mL biotinylated VAMP-2/B (aa33-94), and 100 ng/mL biotinylated VAMP-2/DF (aa34-71) (all obtained from Petra Henklein, Institute for Biochemistry, Charité Berlin) was incubated with BoNT in cleavage buffer (total reaction volume 100 µL/well) for 4 h at 37°C in a hybridisation oven under constant back and forth movement. Reactions were performed in flat bottom 96-well plates. Then, a mixture of four different Luminex bead regions coupled to mAb SNAP/A/291, VAMP/B/151, SNAP/E/1466, or VAMP/F/425 diluted in 1 % BSA/PBS supplemented with 500 mg/mL carboxymethyl (CM)-dextran were added (100 µL/well with 2500 beads/well) to the cleavage reaction and incubated for 90 min at RT on a multiwell plate shaker at 600 rpm. Beads were washed with PBS-T (2 ×, 200 µL/well) and incubated with 2 µg/mL streptavidin conjugated phycoerythrin (Streptavidin-R-Phycoerythrin PJRS34, SA-PE) diluted in 1 % BSA/PBS (100 µL/well) for 30 min at RT on a multiwell plate shaker at 600 rpm. Finally, beads were washed (3 ×, 200 µL/well) diluted in 100 µL 1 % BSA/PBS and samples were analysed using a Bio-Plex 200 system.

# Supporting figures

**Supporting Figure S1.** Reactivity of Neo-mAbs towards neoepitope comprising KLH-coupled peptides, BSA and full-length uncleaved SNARE substrates. Neo-mAbs were characterised by indirect ELISA to demonstrate their exclusive specificity towards the respective neoepitope. 8-mer KLH-coupled peptides representing the end of the C- or N-terminal fragment (indicated as “C” or “N” in parentheses, respectively) generated in SNAP-25 after cleavage by BoNT/A, C and E (Neo-A, Neo-C, Neo-E) or in VAMP-2 after cleavage by BoNT/B, D and F (Neo-B, Neo-D, Neo-F) were coated on microtiter plates along with KLH, full length VAMP-2 or SNAP-25, and BSA and detected by Neo-mAbs (clone name indicated on the top of each panel). Experiments were performed in technical duplicates (Mean ± SD).

**Supporting Figure S2. Performance of all Neo-mAbs in an endopeptidase ELISA.** VAMP-2 or SNAP-25 was coated on microtiter plates, cleaved by serial dilutions of the indicated BoNT serotypes A to F and cleavage products were detected by Neo-mAbs (1 µg/mL, clone names as indicated on the right). Results from two independent experiments each performed in technical duplicates are shown. (n = 4; Mean ± SD). For individual Neo-mAbs recognition of the C- or N-terminal fragment of SNAP-25 or VAMP is indicated after cleavage by BoNT/A to F, respectively. Antibodies depicted bold were selected for implementation into the functional Luminex suspension array.


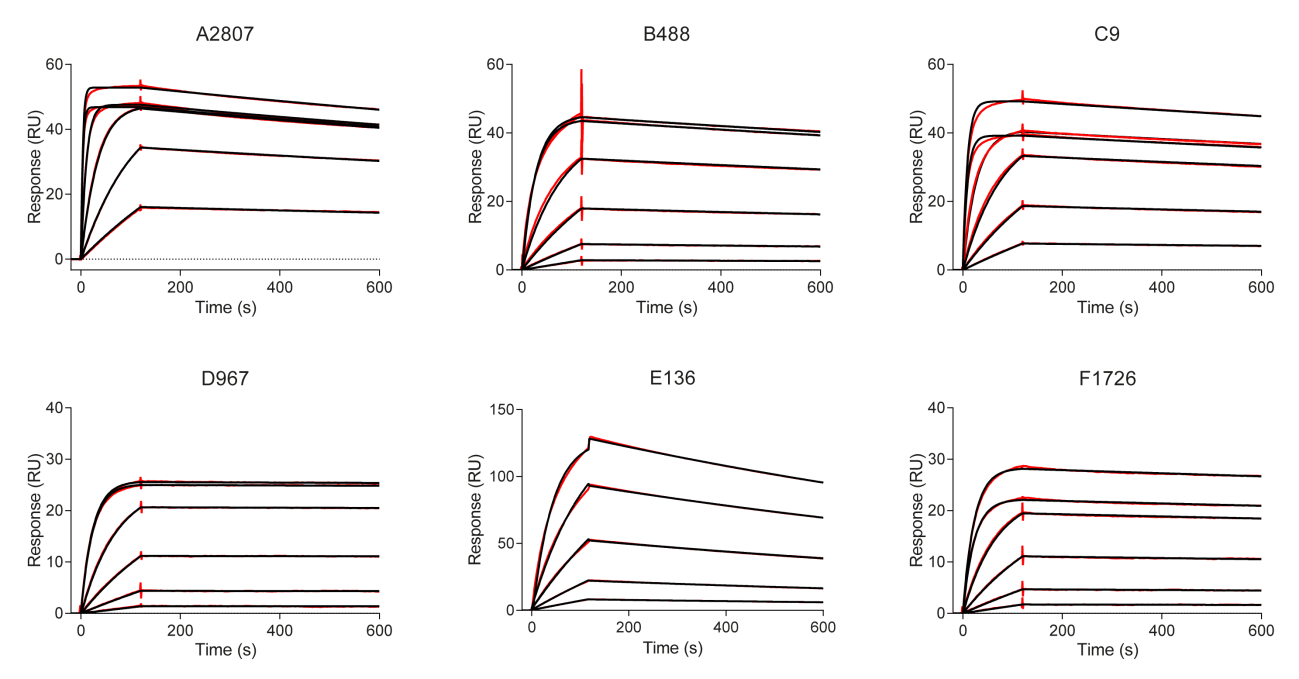


**Supporting Figure S3. SPR measurements of monoclonal anti-BoNT antibodies to determine antibody affinity.** Antibodies were immobilised on a mouse antibody capture chip and serial dilutions of the respective BoNT fragment (Table S1) were injected in a multi-cycle kinetic. Data was fitted using the 1:1 Langmuir binding model (red lines = measured RUs; black lines = fit).

**Table S1.** Binding kinetics and affinity of monoclonal antibodies used for enrichment.

| Antibody | Specificity | *k*_a_ (M^–1^s^–1^) | *k*_d_ (s^–1^) | *K*_D_ (M) | Reference |
| --- | --- | --- | --- | --- | --- |
| A2807 | H_C_A1 | 3.2 × 10^6^ | 2.9 × 10^–4^ | 9.3 × 10^–11^ | ^2^ |
| B488 | H_C_B1 | 3.0 × 10^4^ | 2.1 × 10^–4^ | 7.1 × 10^–9^ | ^2^ |
| C9 | H_C_C | 1.9 × 10^5^ | 1.9 × 10^–4^ | 1.0 × 10^–9^ | ^3^ |
| D967 | H_C_D | 4.2 × 10^5^ | 1.4 × 10^–5^ | 3.3 × 10^–11^ | ^2^ |
| E136 | H_N_E3 | 4.0 × 10^4^ | 5.9 × 10^–4^ | 1.5 × 10^–8^ | ^4^ |
| F1726 | H_C_F1 | 2.2 × 10^6^ | 1.1 × 10^–4^ | 5.0 × 10^–11^ | ^2^ |

**Supporting Figure S4. Lowered sensitivity in a multiplex assay approach due to unspecific binding of VAMP-2 to Luminex beads.** (**a**) Biotinylated SNAP-25 or VAMP-2 was incubated with BoNT/A or BoNT/B, respectively. Cleavage products were detected in a singleplex assay with Neo-mAb SNAP/A/291 or VAMP/B/151 coupled to Luminex beads. When VAMP-2 was present in the reaction mixture (right panel), high unspecific signals appeared, albeit of the added toxin concentration. (**b**) Unspecific binding of VAMP-2 to Luminex beads could be blocked by the addition of carboxymethyl dextran to the reaction mixture. Biotinylated SNAP-25, VAMP-2/B, and VAMP-2/DF was incubated with BoNT/A, B, E or F as indicated. Cleavage products were detected in a multiplex assay with Neo-mAbs (SNAP/A/291, VAMP/B/151, SNAP/E/1466, VAMP/F/425) coupled to Luminex beads diluted in carboxymethyl-dextran.

**Supporting Figure S5. Long cleavage duration increases assay sensitivity exemplified with the duplex assay detecting BoNT/A and B activity.** BoNT/A or B was mixed with VAMP-2 and SNAP-25 coupled to Luminex beads for substrate cleavage for 30 min to 18 h as indicated. Corresponding cleavage products were subsequently detected by Neo-mAbs (detected by mAbs SNAP/A/291 and VAMP/B/1148).

**Supporting Figure S6.** Comparison of results obtained for the duplex-assay detecting BoNT/A and B in two different laboratories. BoNT was diluted in BSA/PBS **(a)** or serum **(b)** and captured via paramagnetic beads coupled to anti-BoNT antibodies. VAMP-2 and SNAP-25 coupled to microspheres were added to captured toxin for substrate cleavage and cleavage products were detected by Neo-mAbs. (**a**) BoNT diluted in BSA/PBS was titrated. (**b**) Toxin recovery of spiked serum samples was determined in two independent laboratories. Results of two independent experiments, each performed in technical duplicates, are shown (n = 4; Mean ± SD; MFI = Median fluorescent intensity).

**Supporting Figure S7.** Testing of toxin spiked serum samples in the three duplex-assays. Different concentrations of BoNT diluted in BSA/PBS (plain bars), or in serum (chequered bars) were detected with the respective duplex-assay (BoNT/A+B **[a]**, C+D **[b]** or E+F **[c]**, please see Figure 3 and Figure 4). Toxin was enriched prior to detection by incubating toxin solutions with magnetic beads coupled to monoclonal BoNT specific antibodies. Captured toxin was added to VAMP-2 or SNAP-25 coupled Luminex microspheres for substrate cleavage and cleavage products were detected by Neo-mAbs. The dashed line indicates the respective LOD (Cut-off: mean + 3.29 × SD of blank values). Results of two independent experiments, each performed in technical duplicates, are shown (n = 4; Mean ± SD; MFI = Median fluorescent intensity).

**Supporting Figure S8.** Detection of BoNT/A and B in different food samples with the duplex-assay. Different concentrations of BoNT/A or B diluted in BSA/PBS (plain bars), or in different foods (fish, beans or sausage extracts as indicated) were detected with the duplex-assay for BoNT/A and B. Toxin was enriched prior to detection by incubating toxin solutions with magnetic beads coupled to monoclonal BoNT specific antibodies. Captured toxin was added to VAMP-2 or SNAP-25 coupled Luminex microspheres for substrate cleavage and cleavage products were detected by Neo-mAbs. The dashed line indicates the respective LOD (Cutoff: mean + 3.29 × SD of blank values). Results of two independent experiments, each performed in technical duplicates are shown. (n = 4; Mean ± SD; MFI = Median fluorescent intensity).

# Supporting references

1 Myszka, D. G. Improving biosensor analysis. *J Mol Recognit* **12**, 279-284, doi:10.1002/(sici)1099-1352(199909/10)12:5<279::aid-jmr473>3.0.co;2-3 (1999).

2 Hansbauer, E. *Stationäre und mobile Verfahren zur Detektion und Differenzierung biologischer Toxine*, Freie Universität Berlin, (2016).

3 Hansbauer, E. M. *et al.* Detection, differentiation, and identification of botulinum neurotoxin serotypes C, CD, D, and DC by highly specific immunoassays and mass spectrometry. *Analyst* **141**, 5281–5297, doi:10.1039/c6an00693k (2016).

4 Simon, S. *et al.* Recommended immunological strategies to screen for botulinum neurotoxin-containing samples. *Toxins (Basel)* **7**, 5011−5034 (2015).
